# Representations of pitch and slow modulation in auditory cortex

**DOI:** 10.3389/fnsys.2013.00062

**Published:** 2013-10-02

**Authors:** Daphne Barker, Christopher J. Plack, Deborah A. Hall

**Affiliations:** ^1^Audiology and Deafness, School of Psychological Sciences, The University of ManchesterManchester, UK; ^2^MRC Institute of Hearing ResearchNottingham, UK; ^3^Nottingham Hearing Biomedical Research Unit, National Institute for Health ResearchNottingham, UK

**Keywords:** pitch, iterated ripple noise, IRN, planum temporale, Heschl's gyrus, spectro-temporal modulation

## Abstract

Iterated ripple noise (IRN) is a type of pitch-evoking stimulus that is commonly used in neuroimaging studies of pitch processing. When contrasted with a spectrally matched Gaussian noise, it is known to produce a consistent response in a region of auditory cortex that includes an area antero-lateral to the primary auditory fields (lateral Heschl's gyrus). The IRN-related response has often been attributed to pitch, although recent evidence suggests that it is more likely driven by slowly varying spectro-temporal modulations not related to pitch. The present functional magnetic resonance imaging (fMRI) study showed that both pitch-related temporal regularity and slow modulations elicited a significantly greater response than a baseline Gaussian noise in an area that has been pre-defined as pitch-responsive. The region was sensitive to both pitch salience and slow modulation salience. The responses to pitch and spectro-temporal modulations interacted in a saturating manner, suggesting that there may be an overlap in the populations of neurons coding these features. However, the interaction may have been influenced by the fact that the two pitch stimuli used (IRN and unresolved harmonic complexes) differed in terms of pitch salience. Finally, the results support previous findings suggesting that the cortical response to IRN is driven in part by slow modulations, not by pitch.

## Introduction

Pitch is the sensation whose variation is associated with musical melodies. It is arguably the most important perceptual feature of music and is one of the main cues in speech and in sound segregation. There are many different physical features that can elicit the same pitch percept. For example, although a middle C played on the piano sounds very different to a middle C played on the guitar or sung, it is still recognized as the same note. It is this phenomenon that has led auditory scientists to postulate the existence of a “pitch center”—a region of auditory cortex responsible for representing pitch, regardless of the physical attributes from which it arises. It has been assumed that such a region would elicit a greater response to pitch stimuli with stronger pitch salience (the strength of the pitch percept) than it would to stimuli with weaker pitch salience (Griffiths et al., 1998; Krumbholz et al., 2003; Penagos et al., 2004; Hall and Plack, 2009; Griffiths, 2001).

Iterated ripple noise (IRN) is a type of pitch-evoking stimulus that is created by generating a sample of noise, imposing a delay, and adding (or subtracting) the delayed version to (or from) the original (Yost, 1996). The delay-and-add process introduces temporal regularity, which evokes a pitch percept that is related to the reciprocal of the delay. The more times this delay-and-add process is repeated, the more salient the pitch becomes (Yost, 1996). The fact that pitch salience can be increased easily by repeating the iterative process has made IRN a popular choice of stimulus for use in neuroimaging studies searching for a pitch center. These studies worked on the subtractive assumption that deducting the activation produced by spectrally matched Gaussian noise from that produced by IRN leaves a representation of the pitch response. The IRN response that has been attributed to pitch is highly consistent across individual listeners and is also reproducible between studies (Patterson et al., 2002; Krumbholz et al., 2003; Seither-Preisler et al., 2004, 2006; Hall et al., 2005, 2006; Hertrich et al., 2005; Barrett and Hall, 2006; Schönwiesner and Zatorre, 2008; Hall and Plack, 2009). Most of these studies have revealed an IRN-related response in an auditory region located antero-lateral to primary auditory cortex, in the lateral portion of Heschl's gyrus (HG), but not restricted to this region. When pitch stimuli other than IRN are used, however, the inter-listener consistency decreases and the group-averaged pitch response appears posterior to lateral HG, in planum temporale (Hall and Plack, 2007, 2009; García et al., 2010; Barker et al., 2011, 2012). Hall and Plack (2009) suggested that the reason for this difference is that IRN contains an additional acoustic feature, not present in other pitch-evoking stimuli, that elicits a greater differential response in lateral HG than other pitch stimuli.

**Figure 1:**
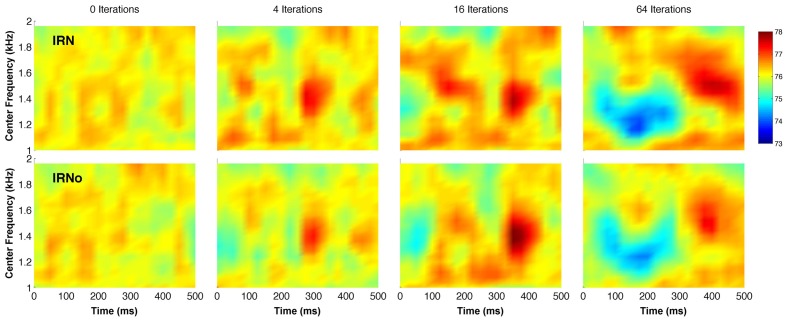
**Simulated cochlear representations of IRN (top row) and IRNo (bottom row) in the form of spectrograms**. The number of delay and add iterations increases from left to right. The analysis smoothes the representation in both time and frequency domains to remove any fine structure related to pitch. All stimuli were created from the same original sample of Gaussian noise, and the IRNo stimuli on the bottom row are processed versions of the stimuli on the top row (IRN). The color bar shows model output in dB SPL. See Barker et al. (2012) for details of the model.

IRN is made from a sample of Gaussian noise, which has rapidly varying envelope fluctuations. However, the iterative delay-and-add process introduces broad spectro-temporal features into the noise (Hall and Plack, 2009) (Figure 1). Most previous pitch studies using IRN have not been designed to separate the pitch response from the response to the slowly varying spectro-temporal fluctuations. In order to determine whether it is the pitch, the slowly varying modulations or an interaction between the two that drives the IRN-related response, Barker et al. (2012) created a new type of stimulus. This novel stimulus consists of IRN that has been processed in a way that removes the temporal fine structure responsible for the pitch percept, whilst leaving the slowly varying spectro-temporal features intact. IRN that is processed in this way is called “no-pitch IRN” (IRNo). Results from psychophysical testing indicate that the perceptual discriminability of IRNo modulations improves with increasing number of iterations, in the same way that pitch discrimination thresholds reduce with increasing iterations for IRN (Barker et al., 2012). This is because the depth of the modulations in IRNo increases with increasing iterations.

Since the strengths of the pitch and modulation percepts appear to covary, the results of studies that have examined the neural response to pitch salience, using IRN as the sole pitch-evoking stimulus are potentially confounded by the response to the slowly varying spectro-temporal modulations (Griffiths et al., 1998; Patterson et al., 2002; Krumbholz et al., 2003; Seither-Preisler et al., 2004, 2006; Barrett and Hall, 2006; Hall et al., 2006; Schönwiesner and Zatorre, 2008). In a previous fMRI study, Barker et al. (2012) demonstrated that the response to these modulations contributes to the cortical auditory response that authors of previous IRN studies have interpreted as pitch specificity. In that study, IRN produced significant activation when contrasted with Gaussian noise, but did not produce any significant activation when contrasted with IRNo. Barker et al. also found that the high inter-listener consistency (75% in the left hemisphere and 88% in the right) decreased by 37% (to 38%) in the left hemisphere and by 44% (to 44%) in the right when IRNo was used as a control compared to when the control was a Gaussian noise.

In the first fMRI study to dissociate the effects of energy onset and pitch onset, García et al. (2010) revealed a non-linear blood oxygenation level dependent (BOLD) response for the addition of one stimulus feature (pitch) to another (energy). The authors suggested that the same neural population was responding to both stimulus features and the sum of the responses reached a maximum saturating limit. If slow-rate modulations and pitch are processed by the same neural population, it is possible that these two features saturate the neural response so that the addition of one feature has little effect on the response to the other. While Barker et al. (2012) found that BOLD responses to IRN and IRNo are broadly similar, suggesting that slow-rate modulations influence the response to IRN, they did not include a stimulus with pitch but without slow modulations, so they were unable to separately manipulate slow modulations and pitch.

The primary motivation for the current study was to quantify the relation between cortical responses to pitch (in general) and to slow-rate spectro-temporal modulations. The research question was examined within a spherical region-of-interest centered anatomically on an *a priori* estimate of the location of the pitch center based on co-ordinates reported in the published literature.

The second research question addressed by the current study concerned the effect of pitch and modulation salience on the BOLD response in the pitch-responsive region. Pitch salience was manipulated in two ways: using IRN with different numbers of iterations and using an unresolved harmonic complex with and without a noise masker. Additionally, IRNo stimuli (with a corresponding number of iterations) were used to determine whether activation increases with increasing modulation depth.

In summary, the main research questions addressed here are:

- Are the responses to slowly varying spectro-temporal modulations and to pitch co-located?
- Are the generators of the pitch and modulation responses sensitive to differing levels of salience for these features?

## Materials and methods

### Listeners

Fourteen listeners (seven males, seven females; age range 22–48 years) with normal hearing (≤20 dB hearing level between 250 Hz and 8 kHz) took part in fMRI testing. All listeners were right-handed (laterality index = 50, Oldfield, 1971). Seven listeners were musically trained between grade 2 and grade 7 (# 02, 07, 12, 19, 22, 23, and 25) while three others reported informal musical experience (self-taught/ungraded, # 05, 09, and 13). None had a history of any neurological or hearing impairment. Listeners gave written informed consent and the study was approved by the Medical School Research Ethics Committee, University of Nottingham. The scanning session for one of the listeners (# 25) had to be terminated due to a significant region of unilateral local MR signal decay around auditory cortex, possibly due to a shimming artifact which could not be rectified. Another subject (# 19) had to be excluded from the analysis because the fMRI time series failed the subjective quality control; the amount of head motion exceeded 3 mm and 3° in at least one plane each, hence there was an absence of reliable sound-related activity.

### Conditions

The experimental design comprised 10 stimulus conditions which part crossed the factors pitch, spectro-temporal modulation, and salience. Two types of pitch-evoking stimuli were employed; IRN and unresolved harmonic complex tones (unres). IRN stimuli comprised three levels of pitch salience (4, 16, and 64 iterations—denoted IRN_4_, IRN_16_, and IRN_64_, respectively), while the unres stimuli had two levels of pitch salience (masked and unmasked unres). Another stimulus contained slowly-varying spectro-temporal fluctuations, but did not evoke a pitch percept (IRNo). This stimulus had three levels of fluctuation salience (4, 16, and 64 iterations—denoted IRNo_4_, IRNo_16_, and IRNo_64_, respectively). The design also included two control conditions. The first was a Gaussian noise (noise) and the second was a Gaussian noise that had been processed in the same way as the IRNo stimuli (processed noise).

### Stimuli

All of the stimuli were matched in their average spectrum (both in spectral range and spectral density) but differed in whether they had a temporal pitch structure or slow spectro-temporal modulations. All IRN and unres stimuli evoked a pitch corresponding to a 100-Hz tone. For the unres conditions, the fundamental frequency (f0) was 100 Hz. Harmonics were added in cosine phase, and the stimuli were bandpass-filtered between 1 and 2 kHz to remove low-numbered harmonics that are resolved (i.e., separated out) by the peripheral auditory system. As in previous studies (e.g., Hall and Plack, 2009) the removal of resolved harmonics was necessary to eliminate tonotopic differences between the pitch stimuli and the control noise, that could have caused differences in activation driven by processes unrelated to pitch. For the unmasked unres condition, the level of each harmonic was 72 dB SPL, chosen so that the gross spectral density (and overall level) of all the stimuli was the same. To make the low-pitch-salience (masked) unres, a bandpass-filtered (1–2 kHz) Gaussian noise masker was added to the unres complex so that the level of the complex tone equaled the level of the masking noise (0 dB signal-to-noise ratio). The level of each harmonic for the masked unres condition was 69 dB SPL, and the spectrum level of the added noise masker was 49 dB SPL, again chosen so that the gross spectral density (and overall level) of all the stimuli was the same. The addition of a noise masker in the spectral region of the unmasked unres reduces the pitch salience. A pilot psychophysical study using nine listeners revealed that f0 discrimination thresholds for masked unres were on average 11% higher than for unmasked unres.

IRN stimuli were generated by a delay-and-add process performed on a Gaussian noise. A delay of 10 ms was imposed before adding the delayed noise back to the original sample. The delay-and-add process was repeated 4, 16 or 64 times to generate the three IRN conditions, and each stimulus was adjusted to a spectrum level of 52 dB SPL. The IRN was bandpass filtered (1–2 kHz) to remove the resolved harmonics.

To create IRNo, a conventional IRN stimulus was generated as above. The IRN was sampled using a rectangular window with a 10-ms duration. A fast Fourier transform (FFT) was used to generate the magnitude and phase spectra of the sample, and the phase of the components was randomized. An inverse FFT was then used to regenerate the time representation. The sampling window was advanced by half of the IRN delay (5 ms) and the process repeated. The processed samples were overlapped and added (preserving the start-times of the samples), and adjusted to a spectrum level of 52 dB SPL. The phase randomization process removes any correlation in the fine structure between samples, obliterating the harmonic structure and the pitch cue. However, the slowly varying broad spectral features are preserved. These fluctuations are visible in the spectrogram representation of IRN when it is smoothed in both time and frequency domains to remove any fine structure (Figure 1). The process was repeated using the IRN_4_, IRN_16_, and IRN_64_ conditions to generate the three IRNo conditions. All experimental stimuli included a noise masker, low-pass filtered at 1 kHz and with a spectrum level of 52 dB SPL, to mask cochlear distortion products.

The noise control had a 52 dB SPL spectrum level and was low-pass filtered at 2 kHz. The processed noise control was generated in the same way as the IRNo, but was otherwise identical to the noise control. The processed noise was perceptually identical to the Gaussian noise but was included to control for any unforeseen effects of processing. All stimuli were matched in bandwidth (0–2 kHz) and spectral density, and hence overall energy (85 dB SPL). Every experimental and control stimulus was gated to produce a time waveform with a 580-ms steady state and 10-ms linear-intensity ramps.

**Figure 2:**
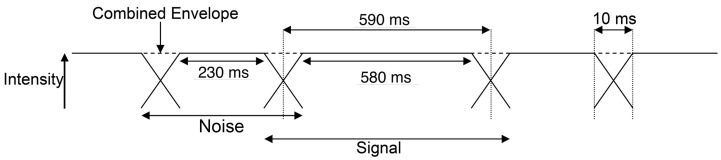
**Schematic representation of the continuous stimulation paradigm used for presentation of stimuli in the MR scanner**. The 10-ms ramps of the two sounds in each sequence were overlapped at the 3 dB SPL point (at 5 ms) to produce a stable envelope.

The energy onset response is an effect that dominates responses in the auditory cortex to repeated bursts of sounds, so that sensitivity to pitch is reduced (Krumbholz et al., 2003; Seither-Preisler et al., 2004; García et al., 2010). To improve sensitivity to the features of interest, we employed a “continuous stimulation” paradigm in which experimental sounds were interspersed by short bursts of noise. In the MR scanner, stimulus conditions each comprised a 15.19-s alternating sequence of 600-ms experimental sounds each separated by 250 ms Gaussian noise (durations included onset and offset ramps) with the same overall spectrum (0–2 kHz) and sound level as the experimental sounds. The 10-ms ramps of the two sounds in each sequence were overlapped at the 3 dB downpoint (at 5 ms); there were 18 presentations of each sound, 19 presentations of each noise and the remaining 5-ms ramps at the beginning and end of the sequence (Figure 2). Sound files of all stimuli have been included as supplementary material.

### fMRI protocol

Scanning was performed on a Philips 3 Tesla Intera Achieva using an 8-channel SENSE receiver head coil. A *T1*-weighted high-resolution (1 mm^3^) anatomical image (*matrix* size = 256 × 256, 160 sagittal slices, *TR* = 8.2 s, *TE* = 3.6 ms) was collected for each subject. The anatomical scan was used to position the functional scan centrally on HG, and care was taken to include the entire superior temporal gyrus and to exclude the eyes. Functional scanning used a *T*2^*^-weighted echo-planar sequence with a voxel size of 3 mm^3^ (*matrix* size = 64 × 64, 32 oblique-axial slices, *TE* = 36 ms). Sparse imaging with a *TR* of 8188 ms and a clustered acquisition time of 1990 ms was used (Edmister et al., 1999; Hall et al., 1999). The beginning of every odd-numbered scan triggered the onset of a stimulus condition, with the even-numbered scans occurring midway through the stimulus and a short pause before the start of the next stimulus. A SENSE factor of 2 was applied to reduce image distortions and a SofTone factor of 2 was used to reduce the background scanner noise level by 9 dB. Functional data was acquired over four runs of 98 scans each. Each sound condition had a total of 32 scans, with 34 scans for the silent baseline. Listeners were requested to listen attentively to the sounds, but were not required to perform any task. A custom-built MR compatible system delivered distortion-free sound using high-quality electrostatic headphones (Sennheiser HE60 with high-voltage amplifier HEV70) with passive noise attenuation. An active noise control (ANC) device (Hall et al., 2009) was used to reduce the overall acoustical scanner noise by a further 14 dB. Eight scans were appended to the beginning of the run in order to initialize the noise cancelling device. These scans were excluded from the analysis.

### Data analysis

**Figure 3:**
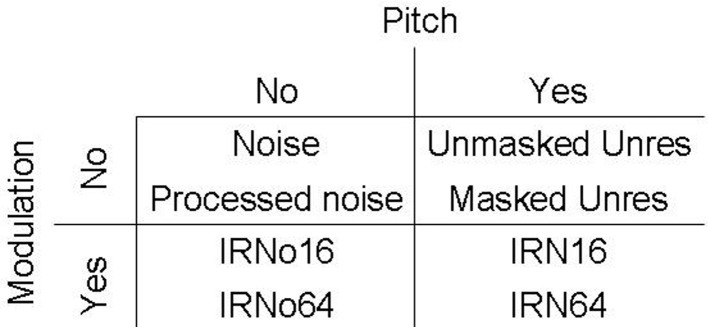
**Schematic representation of the subset of stimuli that contribute to the 2 × 2 factorial design**. Each cell in the matrix contains two levels of salience except for the “no pitch, no modulation” cell.

Images were analyzed separately for each listener using statistical parametric mapping (SPM5, http://www.fil.ion.ucl.ac.uk/spm). Preprocessing steps included realignment to correct for subject motion, normalization of individual scans to a standard image template, and smoothing with a Gaussian filter of 8 mm full width at half maximum (FWHM). The realignment process generated estimates of the scan-to-scan movement for three translations (x, y, and z planes) and three rotations (roll, pitch, and yaw). These were included as variables in the individual design specification in addition to the 10 sound conditions and the four scanning runs. The silent baseline was implicitly modeled in the design. The first-level general linear model assessed the variables of interest with respect to the scan-to-scan variability. A high-pass filter cutoff of 420 s was used to remove low frequency confounds. The resulting model estimated the fit of the design matrix (X) to the data (Y) in each voxel in order to provide β-values (the contribution of a single regressor to the overall fMRI signal). Separate statistical contrasts for each sound condition were specified relative to the silent baseline. To investigate the differential responses across conditions, a One-Way ANOVA was specified at the second level with all 10 sound contrasts, using the preceding contrast images for each individual as input. We defined the model in this way because it provides maximum flexibility for assessing the different effects of interest. Different combinations of contrast weights were then specified from the variables in the ANOVA to determine differences between factors. Contrast weights for each of the stimulus conditions of interest (pitch and slow modulation) were defined to provide a factorial model where two stimulus conditions contributed to each cell in the matrix. The design is represented schematically in Figure 3. It is important to note that the pitch salience of the IRN is not matched to the salience of the unres and so the design is not fully factorial.

Although 14 listeners were scanned, only 12 were included in the analyses (reasons for excluding subjects 19 and 25 were mentioned in the Listeners section above). To improve external validity, our interpretation of the pitch- and modulation-related activity was informed by a spherical region of interest (ROI) with a radius = 10 mm. This ROI was centered on the average peak co-ordinates that had been derived from four previous pitch studies (Table 1) with coordinates *x*-58 *y*-24 *z*-7 in the left hemisphere and *x*-63 *y*-17 *z*-5 in the right. All studies included in the average used normal-hearing participants with no history of neurological disease. Studies using IRN were excluded because of the potential confound with the response to slow modulation and only those studies reporting Montreal Neurological Institute (MNI) coordinates for non-IRN pitch responses could be included. Pitch-related activation within this spherical ROI was interpreted to represent a highly consistent pitch response across studies. This spherical ROI encompassed parts of central and lateral HG and PT. Localization was made with reference to a software toolbox in SPM5 that estimates the cytoarchitectonic subdivisions of HG and assigns probability values estimating the likelihood that a voxel occurs within a particular auditory field (Morosan et al., 2001; Eickhoff et al., 2006). According to this particular anatomical scheme, medial HG is called Te 1.1, central HG is called Te 1.0, and lateral HG is called Te 1.2.

**Table 1:** **Location (MNI coordinates) of pitch-related responses identified by previous fMRI studies using various pitch-evoking stimuli**.

	**Left**			**Right**		
	***x***	***y***	***z***	***x***	***y***	***z***
Hall and Plack, 2009	No left hemisphere clusters			64	−18	4
Puschmann et al., 2010	−50	−20	5	58	−12	7
García et al., 2010 (cHP)	−58	−24	8	64	−16	6
García et al., 2010 (unres)	−62	−24	8	66	−18	6
Barker et al., 2011	−62	−28	8	64	−22	4
Average	−58	−24	7	63	−17	5

Voxels significant at p < 0.05 FDR corrected within the spherical ROI.

Examining the main effect of slow modulation and the interaction between slow modulation and pitch were also restricted to the same spherical region in order to ascertain whether any such effects might be present within the pitch-responsive region. All significant results have been controlled for type I errors by employing a volume correction based on the number of independent voxel elements within the spherical ROI. This correction used a false discovery rate (FDR) threshold of *p* < 0.05 (Genovese et al., 2002).

## Results

### Sensitivity to pitch and to slow modulation

**Figure 4:**
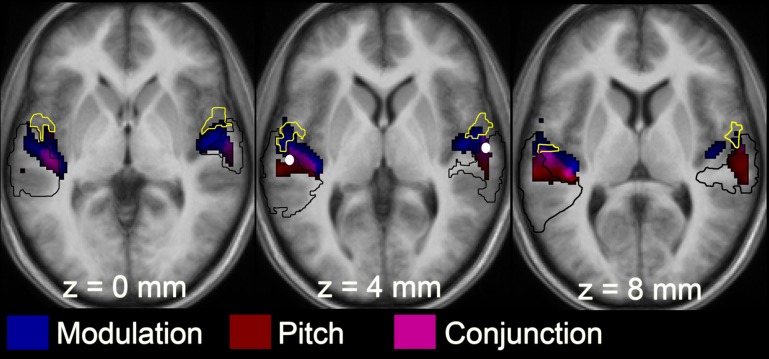
**Statistical *T* map from the 2 × 2 factorial ANOVA showing locations of the group-averaged responses for the main effects of slow modulation (blue) and pitch (red), and a conjunction for the two features (pink)**. The yellow border denotes Te 1.2 (lateral portion of HG) and the black border outlines PT (informed by Westbury et al., 1999). Activation is overlaid onto an average anatomical image made from the 12 individual listeners. The left hemisphere is on the left-hand side of each anatomical image. These images used an uncorrected threshold *p* < 0.05. This figure demonstrates the patterns of activation across the entire cortex, although the analyses were restricted to a 10-mm sphere centered on the white spots in the middle panel.

To determine whether the responses to pitch and to slow modulation are co-located to the same voxels within the pitch-related ROI, the response to pitch was measured by comparing the four most salient pitch conditions (masked unres, unmasked unres, IRN_16_, IRN_64_) to the four matched no-pitch conditions (noise, processed noise, IRNo_16_ and IRNo_64_) (Figure 4). Within the spherical ROI, this contrast highlighted bilateral peaks of pitch-related activity with maxima in posterior auditory cortex (PT) (*x*-64 *y*-28 *z*-6 in the left hemisphere and *x*-64 *y*-22 *z*-10 in the right, Table 2). The cluster in the left hemisphere contained two further maxima. Probability estimates placed both peaks in central HG, although one was potentially within lateral HG. The right hemisphere cluster contained one further maximum. This peak was most likely in PT, although again lateral HG could not be ruled out.

**Table 2:** **Location (MNI coordinates) of the effects of pitch and modulation, and of the conjunction between pitch and modulation**.

	**Peak**	**Left**				**Right**			
		***x***	***y***	***z***	***n***	***x***	***y***	***z***	***n***
Main effect of	1	−64	−28	6	320	64	−22	10	156
Pitch	2	−54	−20	8		62	−6	4	
	3	−50	−20	2					
Main effect of	1	−58	−14	4	228	64	−10	2	187
modulation	2	−52	−18	0		62	−8	2	
	3	−64	−26	10		62	−6	4	
	4					56	−10	−2	
	5					56	−8	2	
Conjunction	1	−56	−20	8	171	64	−12	4	87
	2	−50	−20	2		62	−5	4	
	3	−64	−26	10					

Voxels significant at p < 0.05 FDR corrected within the spherical ROI. n, indicates the number of voxels within each cluster.

The main effect of slow modulation was determined by contrasting IRNo_16_, IRNo_64_, IRN_16_, and IRN_64_ with noise, processed noise, masked and unmasked unres (Figure 4). This contrast did not reveal any clusters of activity that survived correction for multiple comparisons (FDR *p* > 0.05).

**Figure 5:**
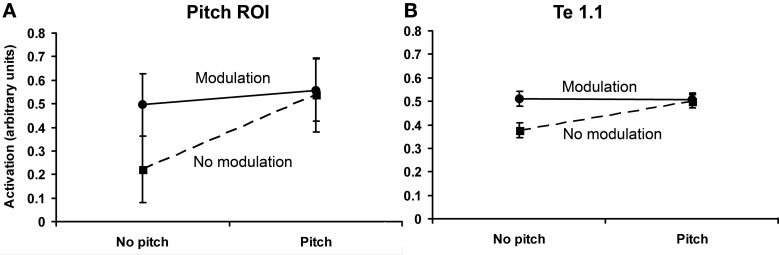
**Plot of the results of the 2 × 2 factorial design within the pitch-responsive ROI (A) and within medial HG (B)**. The ordinate measures percentage increase in BOLD activation from baseline. Error bars show standard errors.

Although the random effects analysis did not suggest a significant effect of slow modulation, this voxel-by-voxel analysis approach is rather conservative. For example, statistical significance is dependent upon the response being present in the same voxel location across listeners. To allow for some degree of spatial variability, we conducted a region-based analysis averaging each condition-specific response (i.e., mean β-values) across all voxels within the spherical ROI, separately for each listener. Data extraction for the region-based analysis used the approach described by Hall and Plack (2009). To determine the effects of pitch and modulation and the nature of any interaction between these two factors within the spherical ROI, values were averaged for the two conditions in each cell of the 2 × 2 ANOVA depicted in Figure 3. The region-based analysis revealed significant effects of both pitch [*F*_(1, 11)_ = 24.30, *p* < 0.05] and slow modulation [*F*_(1, 11)_ = 24.19, *p* < 0.05], with a significant interaction between pitch and slow modulation [*F*_(1, 11)_ = 24.55, *p* < 0.05]. The nature of the interaction was such that both of the stimuli that contained slowly varying modulation (IRN and IRNo) and the unresolved harmonic complex elicited a similar response, whereas the stimulus that contained neither pitch nor slow modulation (noise) elicited a lesser response (Figure 5A). The relation between the responses to pitch and slow modulation in the pitch-responsive region is *saturating*: the effects of the two features are not linearly additive. However, it is possible that this interaction was influenced by the weaker pitch salience of IRN compared to the unresolved complex tones (see Section Salience-Related Activity). Although there is no evidence that the responses to pitch and to slow modulation are co-located at the voxel level, it is apparent from the spherical ROI analysis that there is an overall effect of both pitch and slow modulation within that region.

The same analysis performed in medial HG (Te 1.1) also revealed a significant effect of pitch [*F*_(1, 11)_ = 2.76, *p* < 0.05], of slow modulation [*F*_(1, 11)_ = 2.29, *p* < 0.05] and a significant interaction between pitch and modulation [*F*_(1, 11)_ = 34.23, *p* < 0.05]. However, the effects of pitch, and the interaction between pitch and modulation, were smaller in Te 1.1 than in the pitch-related ROI (Figure 5B). Combining the results, there was a significant two-way interaction between pitch and region (pitch-related ROI and Te 1.1) [*F*_(1, 11)_ = 29.92, *p* < 0.05], and a significant three-way interaction between pitch, modulation, and region [*F*_(1, 11)_ = 13.63, *p* < 0.05]. Hence, there is evidence for some regional specificity in the pitch response, and that the response in the pitch-related ROI is not just a generic response to acoustic change.

In order to determine whether IRN-related activity is driven by slowly varying spectro-temporal modulation or by pitch, a 2 × 3 repeated-measures ANOVA was performed within the original spherical ROI for the IRN and IRNo conditions with stimulus (IRN and IRNo) and salience (4, 16, and 64 iterations) as factors. This ANOVA did not reveal a significant effect of stimulus [*F*_(1, 11)_ = 0.981, *p* > 0.05], but there was a significant effect of salience (number of iterations) [*F*_(1.35, 14.87)_ = 9.070, *p* < 0.05 (Greenhouse-Geisser corrected)] with no significant interaction between stimulus and salience [*F*_(2, 22)_ = 2.749, *p* > 0.05]. This pattern of results is consistent with our previous interpretation that the spectro-temporal modulations, not the pitch, drive the IRN-related response (Barker et al., 2012).

### Salience-related activity

The final analyses addressed the second research question: Are the generators of the pitch and modulation responses sensitive to differing levels of salience for these features? A pilot exploration using eight listeners demonstrated that pitch discrimination thresholds for high-salience IRN stimuli were considerably higher than for the low-salience unres stimuli (mean geometric threshold for IRN_16_ and masked unres were 96.9 and 26.4 Hz, respectively [*T*_(1, 7)_ = 4.41, *p* < 0.05]). This finding implies that IRN stimuli elicited a weaker pitch percept than unres stimuli and that the factorial design is not balanced for pitch salience, so these pitch comparisons were analyzed separately. Since the research question relates to an effect of salience *within a pitch-responsive region*, the spherical ROI described previously was applied. For unres stimuli, the subtraction (unmasked unres—masked unres) examined the effect of pitch salience. Within the spherical ROI, this contrast highlighted bilateral clusters in auditory cortex, with peaks located in PT (*x*-58 *y*-30 *z*-8 in the left hemisphere and *x*-60 *y*-22 *z*-6 in the right). The left cluster contained four maxima, of which one was potentially located within lateral HG (*x*-56 *y*-18 *z*-10). The cluster in the right hemisphere contained three maxima including one that incorporated part of lateral HG (*x*-62 *y*-6 *z*-4). To investigate the effect of pitch salience for the IRN stimuli, the subtraction (IRN_64_—IRN_4_) was performed and the results were displayed using an “exclusive mask” for the subtraction (IRNo_64_—IRNo_4_) which means that any voxels showing a differential response to the depth of the spectro-temporal modulations were excluded. There were no maxima for salience-related activity for IRN that remained significant when corrected for multiple comparisons (FDR, *p* > 0.05).

**Figure 6:**
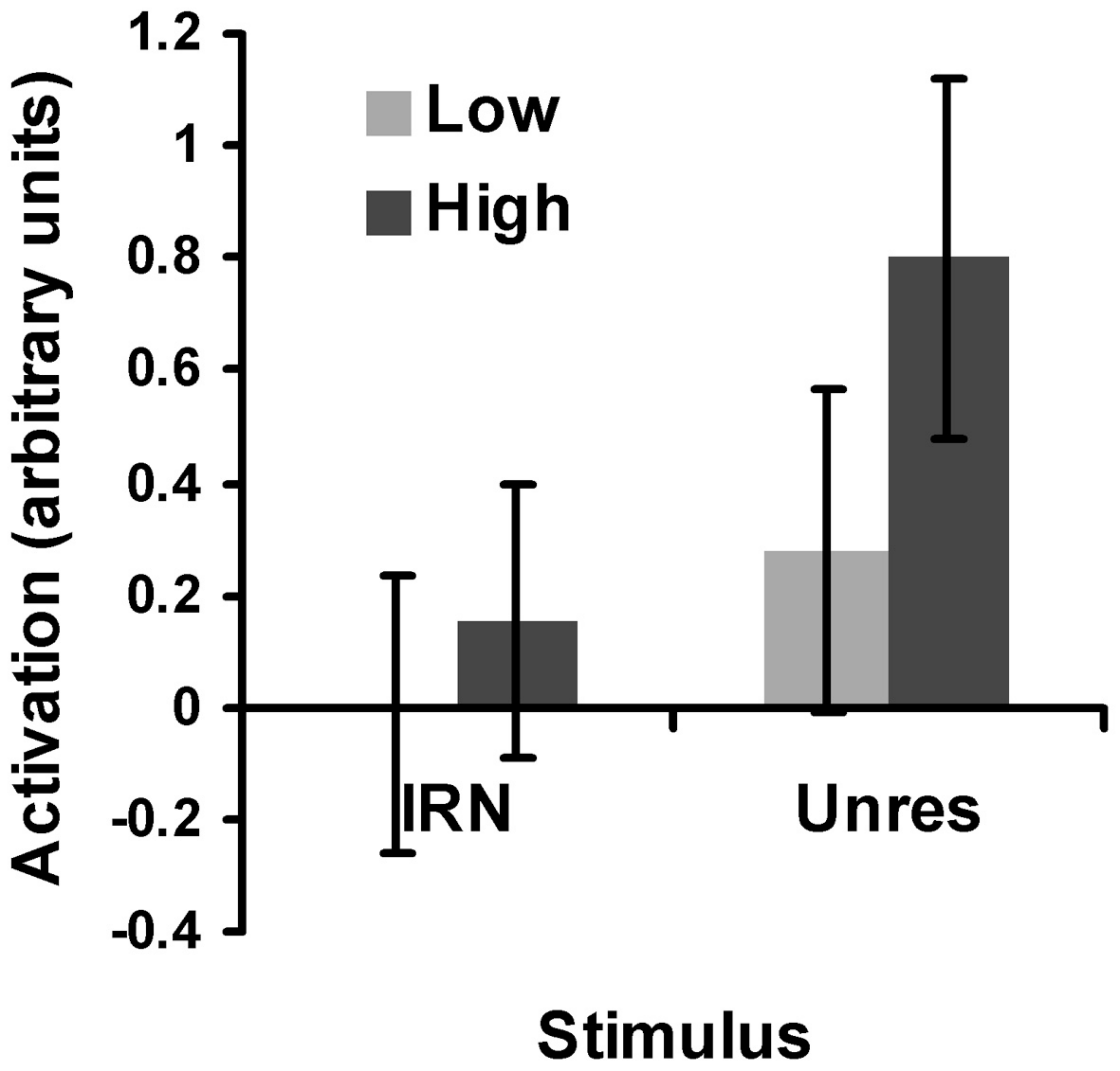
**Plot of the salience analysis results for IRN and for unres within the spherical pitch ROI**. “Activation” refers to the average beta weights: a numerical measure of the effect size. The low salience conditions are represented by the light gray bars and the high salience conditions are represented by the dark gray bars. For the low-salience IRN condition, IRNo_4_ has been subtracted from IRN_4_ and for the high-salience condition, IRNo_64_ has been subtracted from IRN_64_ to remove the effects of slow modulation. Error bars represent 95% confidence intervals.

In order to determine whether the pitch region as a whole was sensitive to pitch salience, the region-based analysis described in 3.1 was performed separately for IRN and for unres. For the IRN stimuli, IRN_64_ and IRN_4_ were contrasted, with values for IRNo_64_ and IRNo_4_, respectively, subtracted to control for the effects of slow-rate modulation. This analysis revealed a significant effect of salience [*F*_(1, 11)_ = 7.84, *p* < 0.05] within the spherical ROI (Figure 6). For unres stimuli, masked and unmasked unres were contrasted. Unsurprisingly (based on the results reported above), this analysis also revealed a significant effect of salience [*F*_(1, 11)_ = 63.02, *p* < 0.05] (Figure 6). It is apparent from Figure 6 that the low-salience unres produced greater activation than the high-salience IRN [*F*_(1, 11)_ = 33.92, *p* < 0.05], which is consistent with results from the psychophysical testing and could explain why there were no significant salience-related voxels for IRN.

To investigate the effect of modulation salience, the subtraction (IRNo_64_ - IRNo_4_) was performed. This contrast did not reveal any supra-threshold clusters (FDR, *p* > 0.05). However, results from the ROI analysis suggest a significantly greater average response to IRNo_64_ than to IRNo_4_ within the spherical ROI [*F*_(1, 11)_ = 5.08, *p* < 0.05]. Hence, when the average BOLD response is taken across all voxels within the pitch-responsive region defined in this study, the region demonstrates sensitivity to both pitch salience and salience of slow-rate modulations.

## Discussion

### Responses to pitch and slow modulation

A previous study (Barker et al., 2012) revealed that the slowly varying spectro-temporal modulations created by the delay-and-add iterative process influence the IRN response, but the results could not determine the precise nature of this influence. It is possible that, due to non-linearities in the neural response (Sidtis et al., 1999; Friston et al., 2000; Devor et al., 2003) there is a saturating interaction between the responses to pitch and to slow modulation whereby the BOLD response is dominated by one feature (e.g., spectro-temporal modulation) so that the response to any additional feature (e.g., pitch) is limited by the saturation of the BOLD signal. In a meta analysis focusing on the role of PT (Griffiths and Warren, 2002), the effects of both pitch (tone vs. noise contrast) and modulation (frequency-modulated vs. unmodulated tone contrast) fell within the spherical ROI used in the current study. It is therefore plausible that either the same populations of neurons are responsible for processing both features, or that there are dispersed feature-specific neurons for pitch and for slow modulation that occupy the same region of auditory cortex. Our ROI analysis constrained our hypothesis to a circumscribed focal brain region, and so we cannot rule out this latter alternative explanation.

The present results revealed a saturating interaction between the responses to pitch and to slow-modulation. This could reflect a saturation in the neural response due to co-location of the representation of the two features. However, a psychophysical pilot experiment revealed that IRN stimuli elicited a much weaker pitch percept than unres stimuli, even when the unres stimuli were masked to reduce the signal-to-noise ratio. With that in mind, it is possible that the pitch percept elicited by IRN was not strong enough to increase the BOLD signal significantly above that of the IRNo stimuli. In other words, we cannot rule out the possibility that the saturating interaction was due to the differing salience of the pitch-evoking stimuli, rather than a saturation in the neural response.

### The IRN response may be driven in part by slow modulations

Due to the lack of a significant difference between the responses to IRN and IRNo discussed above, it is not clear whether the response to IRN is driven mainly by slowly varying modulations or by pitch. This is consistent with the finding of Barker et al. (2012), who reported broadly similar response patterns for IRN and for IRNo within central and lateral HG and within PT. However, both studies indicated a small additional effect of the pitch in IRN over and above the modulation response elicited by IRNo. In the current study, there were significant clusters of activation for the high vs. low salience IRN contrast at an uncorrected level but not for the equivalent IRNo contrast. In the Barker et al. (2012) study, there was a significant linear trend for number of iterations for IRN, but not for IRNo. Furthermore, the contrast (IRN - IRNo) in that study revealed a bilateral pitch-related response for IRN that was co-located for up to seven of their 16 listeners. Therefore, although it appears that slow modulation accounts for the majority of the IRN response magnitude, there is some evidence that pitch does contribute in a small way.

### The pitch-responsive region is sensitive to pitch salience and to modulation salience

Some previous research has suggested a sensitivity to pitch salience in auditory cortex (Griffiths et al., 1998; Penagos et al., 2004; Bendor and Wang, 2005; Gutschalk et al., 2007), although this finding is not universal. For example, using pulse trains with different amounts of jitter, and unresolved harmonic complexes with different relative phases, Barker et al. (2011) actually found a *decrease* in activation with increasing pitch salience. The pitch-evoking stimuli used here contained only unresolved harmonics and as such, they elicit a less salient pitch percept than stimuli containing resolved harmonics (Houtsma and Smurzynski, 1990). However, the unresolved stimuli were sufficiently salient to investigate the relative salience between the different conditions. Results from the current experiment provide evidence for a general sensitivity to pitch salience within the pitch-responsive region, with specific foci that exhibit a strong salience-related response for salient pitch stimuli. As previously mentioned, the pitch of the high-salience IRN condition was not as strong as the pitch of the low-salience unres condition. Hence, it is possible that the high-salience IRN condition was not sufficiently salient to produce an increase in the magnitude of the BOLD response that was large enough to survive correction in the salience-responsive foci for IRN. Indeed, Figure 6 provides support for this conjecture. However, results from the ROI analysis suggest a general sensitivity to differences in salience even for stimuli that evoke a weak pitch percept.

To summarize, the results of the salience analyses suggest that the cortical representation of pitch is sensitive to differing levels of pitch salience. The analysis also provides evidence that the cortical response is sensitive to differing depths of slow modulation, which suggests that slow modulation may affect the salience response for IRN.

### Implications for the location of the “pitch center”

To minimize the effects of onset energy, we chose to use a paradigm in which noise was interleaved between stimuli. As a result of this design choice, all of the conditions except the Gaussian noise condition had perceptible acoustic changes from stimulus to stimulus, and thus the observed response pattern could possibly be driven, at least in part, by a generic response to presence of acoustic changes, rather than to the presence of modulation or pitch *per se*. However, the ROI studied here has been identified by several studies as being selective for pitch using a pulsed paradigm (without interleaved noise) (Hall and Plack, 2009; Barker et al., 2011; Puschmann et al., 2010; García et al., 2010). There is external evidence, therefore, that the ROI is not a region that responds non-specifically to any stimulus change. In addition, in the present study pitch-related activation was greater in the spherical ROI than in Te 1.1, suggesting a specificity for pitch processing in the spherical ROI.

Evidence from MEG and fMRI studies also suggests that the present results probably reflect a specific response to the effects of interest (pitch and slow modulation) rather than to non-specific response to *any* change in stimulus feature. For example, using IRN as their pitch stimulus, Krumbholz et al. (2003) reported a significant magnetic deflection for the change from noise to pitch with no corresponding deflection for the change from pitch to noise. Chait et al. (2006) found distinct temporal and spatial differences between the change from noise to pitch (Huggins pitch and pure tone in noise) and the change from decorrelated to correlated noise. Dipole source modeling locates the responses from both MEG studies in lateral HG, although the spatial resolution is not accurate enough to rule out the possibility that their responses could have been located in PT or across the two regions. Finally, in an fMRI study using Huggins pitch and an unresolved harmonic complex as their stimuli, García et al. (2010) reported a significant difference between their pitch-in-noise vs. constant noise contrast and their noise-in-silence vs. constant noise contrast in the region of PT. However, even if the pitch-sensitive region examined in the present experiment does not respond to all stimulus features, it clearly responds strongly to slow modulations, and the response to these features interacts with the response to pitch. This raises doubts regarding whether the “pitch center” is exclusive for pitch processing.

Within the spherical ROI, it is conjectured that the precise location of pitch-sensitive responses had some spatial variability across individual listeners. Our evidence here is based on the fact that there was no significant voxel-by-voxel response, possibly due to the lack of a voxel-level overlap.

## Summary

The pre-defined pitch-responsive region was found to contain representations for both pitch and slow modulation. There was also a response to pitch salience and to modulation salience in this region. The results support the suggestion made by Barker et al. (2012) that the slowly varying spectro-temporal modulations in IRN affect the response. This finding implies that future studies using IRN as a pitch-evoking stimulus should employ a baseline that controls for these modulations (such as IRNo) and that interpretations from results of previous studies using IRN as their sole pitch-evoking stimulus should be carefully reconsidered.

### Conflict of interest statement

The authors declare that the research was conducted in the absence of any commercial or financial relationships that could be construed as a potential conflict of interest.

## Supplementary material

Audio 1 **An example of the IRN_4_ stimulus used during scanning**.

Audio 2 **An example of the IRN_16_ stimulus used during scanning**.

Audio 3 **An example of the IRN_64_ stimulus used during scanning**.

Audio 4 **An example of the IRNo_4_ stimulus used during scanning**.

Audio 5 **An example of the IRNo_16_ stimulus used during scanning**.

Audio 6 **An example of the IRNo_64_ stimulus used during scanning**.

Audio 7 **An example of the control noise stimulus used during scanning**.

Audio 8 **An example of the masked unresolved harmonic complex used during scanning**.

Audio 9 **An example of the unmasked unresolved harmonic complex used during scanning**.
